# Genetic Determinants Analysis of PB1 and PB2 Genes in H5N1 Avian Influenza Strains from Romania

**DOI:** 10.3390/pathogens15070768

**Published:** 2026-07-21

**Authors:** Maria Rodica Gurău, Florica Bărbuceanu, Alexandru Gligor, Cosmin Șonea, Georgeta Ștefan, Doina Daneș, Elena Negru, Iosif Nicodim Fiț, Kalmár Zsuzsa, Doru Valentin Hristescu, Vlad Barbu Vuță, Raluca Burlacu, Ionica Iancu, Viorel Herman, Stelian Bărăităreanu

**Affiliations:** 1 Faculty of Veterinary Medicine, University of Agronomical Sciences and Veterinary Medicine, 050097 Bucharest, Romania; maria.gurau@fmvb.usamv.ro (M.R.G.); cosmin.sonea@fmvb.usamv.ro (C.Ș.); georgeta.stefan@fmvb.usamv.ro (G.Ș.); doina.danes@fmvb.usamv.ro (D.D.); elena.negru@fmvb.usamv.ro (E.N.); stelian.baraitareanu@fmvb.usamv.ro (S.B.); 2 Institute for Diagnosis and Animal Health (IDAH), 050557 Bucharest, Romania; doru.hristescu@idah.ro (D.V.H.); vlad.vuta@idah.ro (V.B.V.); raluca.burlacu@idah.ro (R.B.); 3 Faculty of Veterinary Medicine, University of Life Sciences “King Mihai I” from Timişoara, 300645 Timişoara, Romaniaviorel.herman@fmvt.ro (V.H.); 4 Faculty of Veterinary Medicine, University of Agriculture Sciences and Veterinary Medicine, 400372 Cluj Napoca, Romania; nfit@usamvcluj.ro (I.N.F.); zsuzsa.kalmar@usamvcluj.ro (K.Z.); 5 Academy of Romanian Scientists (AOSR), Str. Ilfov, Nr. 3, Sector 5, 050044 Bucharest, Romania

**Keywords:** H5N1, avian influenza, PB1, PB2, phylogenetic analysis, molecular epidemiology

## Abstract

Avian influenza is a highly contagious viral disease affecting domestic and wild birds, caused by influenza A viruses of the family Orthomyxoviridae. Continuous genetic surveillance is essential for monitoring viral evolution and identifying molecular determinants associated with pathogenicity and host adaptation. In this study, the PB1 and PB2 polymerase genes of highly pathogenic avian influenza (HPAI) H5N1 viruses detected in Romania between 2017 and 2024 were genetically characterized. A total of 18 organ samples collected from domestic and wild birds in several Romanian counties were analyzed by real-time RT-PCR, followed by conventional RT-PCR, Sanger sequencing, mutational analysis, and phylogenetic reconstruction. High-quality sequence data were obtained for six PB1 and eight PB2 gene fragments. Sequence analysis revealed a high degree of conservation within the PB1 gene, particularly across regions involved in viral replication, indicating strong evolutionary constraints. In contrast, the PB2 gene exhibited several amino acid substitutions, including changes at positions 464 and 678 in selected isolates. Notably, all analyzed strains lacked the mammalian adaptation marker E627K. Phylogenetic analysis clustered the Romanian isolates within Eurasian H5N1 lineages and demonstrated close genetic relationships with contemporary strains circulating in Eastern Europe and Russia. These findings highlight the genetic stability of PB1, the ongoing diversification of PB2, and the importance of continuous molecular surveillance for the early detection of viral variants with potential implications for host adaptation and zoonotic risk.

## 1. Introduction

Avian influenza is an infectious, highly contagious disease caused by single-stranded RNA viruses belonging to the species *Alphainfluenzavirus influenzae* (genus *Alphainfluenzavirus*, family *Orthomyxoviridae*) [1]. The viral particles possess an outer membrane with two main surface antigenic glycoproteins, hemagglutinin (HA) and neuraminidase (NA), which provide subtype and antigenic variant specificity [2,3]. Avian influenza viruses are systematically classified into two distinct groups based on their pathogenicity in host bird populations, as determined via the intravenous pathogenicity index (IPI) test: highly pathogenic avian influenza (HPAI) viruses and low-pathogenic avian influenza viruses (LPAI) [4,5]. Among the several subtypes that continue to menace avian biosecurity and human health are the highly pathogenic avian influenza (HPAI) viruses of the H5 subtype [6]. Human infection is most typically characterized by moderate localized symptoms, such as conjunctivitis or mild respiratory disease. However, sporadic zoonotic transfer of HPAI viruses from infected poultry to people can result in severe clinical consequences and public health emergencies [7]. Among many circulating HPAIV lineages, H5N1 and H5N8 viruses are known to be highly pathogenic with extremely high fatality rates in both birds and mammals, including humans [8].

In recent years, clade 2.3.4.4b H5Nx viruses have become the predominant highly pathogenic avian influenza viruses circulating in Europe, causing unprecedented epizootics in wild birds and poultry. Their continuous geographic expansion, frequent reassortment events, and occasional spillover into mammals have reinforced the importance of molecular surveillance focused on viral genes involved in replication efficiency and host adaptation [4,5,6,7,8].

Avian influenza has been reported in Romania for several decades, with sporadic historical outbreaks followed by major epidemic waves after 2005. Since then, repeated incursions of highly pathogenic H5 viruses have occurred in both domestic poultry and wild birds, reflecting the broader epidemiological dynamics observed across Europe. More recently, clade 2.3.4.4b H5N1 viruses have become established as the dominant lineage responsible for outbreaks throughout the region [9,10].

During the 2005–2014 decade, a cumulative total of 152 outbreaks were recorded across Romania [11]. This emphasizes the need for long-term datasets to uncover regional introduction trends [12]. In the recent decade, a number of viral subtypes such as H5N1, H5N8, H5N5 and reassortant H5Nx viruses have been circulating in the region, contributing to the epidemiology of avian influenza [13,14].

Since 2021, Romania has experienced repeated incursions of highly pathogenic avian influenza viruses belonging to clade 2.3.4.4b, consistent with the broader European panzootic [15,16]. The virus has been detected in both wild birds and domestic poultry, with outbreaks reported in several counties across the country [16]. The isolates analyzed in the present study, collected between 2017 and 2024 from multiple Romanian counties, encompass different epidemic periods and host species, providing an opportunity to investigate the molecular evolution of H5N1 viruses circulating in Romania over time.

Domestic detection may reflect either the persistence of the virus in the area or its local reintroduction in the subsequent year [15]. The H5N1 strains actively reported across Europe, including those from neighboring borders, belong predominantly to clade 2.3.4.4b, which is notorious for generating expansive outbreaks and is heavily characterized by its evolutionary capacity to accumulate mutations [15,16,17,18].

The genome of the HPAI H5N1 virus consists of eight distinct segments of negative-sense single-stranded RNA, with a total length spanning around 13.5 kilobases. Each segment encodes specific proteins that play critical roles in the viral life cycle. These proteins include basic polymerase 1 (PB1, 757 amino acids), basic polymerase 2 (PB2, 759 amino acids), acidic polymerase (PA, 716 amino acids), hemagglutinin (HA, 568 amino acids), nucleoprotein (NP, 498 amino acids), neuraminidase (NA, 499 amino acids), matrix protein 1 (M1, 252 amino acids), matrix protein 2 (M2, 97 amino acids), as well as the non-structural proteins NS1 (225 amino acids) and NS2 (121 amino acids) [19,20,21].

The PB1 gene encodes one of three main components of the heterotrimeric viral polymerase complex that directly mediates transcription and replication of the viral RNA genome. Functioning as the enzymatic “engine” of the replication machinery, PB1 directly catalyzes the elongation of new RNA chains. In addition to this primary role, the PB1 segment can express critical accessory proteins through alternative reading frames, such as PB1-F2 and PB1-N40. PB1-F2 is heavily involved in triggering host cell apoptosis and modulating the cellular immune response, thereby contributing to increased severity of infection. Meanwhile, PB1-N40, a truncated variant of the protein, regulates the precise balance of viral replication processes. Point mutations within the PB1 sequence can influence virus multiplication efficiency, host adaptation kinetics, and overall virulence. In avian strains with zoonotic potential, such as H5N1, structural shifts in PB1 are associated with enhanced adaptation to mammalian cellular environments and more severe clinical manifestations. Consequently, PB1 possesses a dual epidemiological importance: it is vital for the biological life cycle of the virus and serves as a major genetic determinant of pathogenicity and interspecies spillover risks to humans [4]. Within the literature, several recurrent mutations in the PB1 protein have been associated with increased virulence and mammalian adaptation, including 473L, 524T, 678N, 739V, and the critical N66S substitution located inside the accessory PB1-F2 protein.

The PB2 gene encodes one of the essential subunits of the viral RNA polymerase complex of avian influenza A viruses and plays a key role in viral genome replication, transcription, and host adaptation [22]. Together with the PB1 and PA subunits, PB2 contributes to the cap-snatching mechanism required for viral mRNA synthesis, enabling efficient replication within host cells [23,24]. Among the influenza A virus genes, PB2 is considered a major determinant of host specificity and interspecies transmission, as specific amino acid substitutions can enhance polymerase activity and facilitate adaptation to mammalian hosts [25]. The E627K mutation represents one of the most extensively characterized adaptive markers, being associated with increased polymerase efficiency in mammalian cells and enhanced replication at lower temperatures found in the upper respiratory tract of mammals. This mutation has been frequently identified in mammalian-adapted avian influenza strains, including human H5N1 and H7N9 isolates [25,26,27,28,29]. Another important adaptive substitution, D701N, has been linked to improved viral replication and increased interaction with mammalian cellular factors, contributing to enhanced host adaptation and pathogenicity in experimental models [30,31]. Other PB2 mutations, such as Q591R/K, T271A, and G590S/Q591R, have also been associated with mammalian adaptation, highlighting the evolutionary flexibility of the virus in overcoming host barriers [32,33,34]. Therefore, monitoring PB2 genetic variation remains an important component of avian influenza surveillance, as the emergence of adaptive mutations may indicate increased potential for mammalian transmission and future zoonotic risk [35,36]. The objective of this research was to perform a molecular characterization of partial PB1 and PB2 sequences from H5N1 Avian Influenza Virus strains circulating in Romania. The analysis focuses on identifying specific mutational patterns associated with zoonotic risk and establishing molecular links with relevant global sequences.

## 2. Materials and Methods

### 2.1. Animals and Biological Samples

The biological matrices analyzed in this study comprised various organ tissues (including brain, trachea, lungs, spleen, liver, pancreas, kidneys, heart, and intestine) collected from dead domestic and wild birds suspected of or officially confirmed with highly pathogenic avian influenza (HPAI) infection.

During the period 2017–2024, Romania officially notified the National Veterinary Sanitary and Food Safety Authority (ANSVSA) and the World Organization for Animal Health (WOAH) of several waves of highly pathogenic avian influenza (HPAI) outbreaks, most of which were caused by the H5N1 and H5N8 subtypes.

The specific H5N1 outbreaks declared during this interval were in number of 8 and were particularly concentrated at the end of the analyzed period (2021–2024), which explains the number of strains. The focus was on H5N1, because the objective of the investigation was to identify the mutations associated with zoonotic risk in the sequences significant for this phenomenon, which are PB1 and PB 2.

A total of 18 biological samples were collected between 2017 and 2024 from strategic regional nodes in Southeastern Romania, specifically from Brăila, Galați, Ialomița, and Constanța counties (Table 1).

These samples were processed and supplied by the Institute for Diagnosis and Animal Health (IDAH), Bucharest, adhering strictly to institutional biosecurity frameworks and specialized transport conditions mandated for high-consequence avian pathogens.

The technical protocols for field sampling, biosafety packaging, sample identification, and secure transport to the reference laboratory were executed in strict compliance with national legislative frameworks, including Order 145/21.09.2018, and aligned with the Sanitary and Veterinary Norm on control measures for avian influenza (Order 54/28.02.2007) alongside the National Contingency Plan for highly pathogenic avian influenza.

### 2.2. RNA Extraction

Total viral RNA extraction from tissue homogenates was conducted by the Institute for Diagnosis and Animal Health (IDAH), utilizing QIAamp cador Pathogen Mini Kit (Qiagen, Hilden, Germany) according to the manufacturer’s operational instructions. The harvested viral RNA eluates were immediately stored at −80 °C to maintain structural integrity and prevent enzymatic degradation prior to downstream reverse transcription and molecular amplification steps.

### 2.3. Real-Time PCR Method

For primary screening and confirmatory diagnosis, all biological extracts were tested using qualitative Real-Time Reverse Transcription Polymerase Chain Reaction (Real-Time RT-PCR). Commercially available diagnostic kits from Genetic PCR Solutions™ were employed, specifically targeting the Avian Influenza Type A matrix gene, subtype H5, and neuraminidase N1 architectures according to the manufacturer’s product inserts. To ensure analytical rigorousness, all clinical extracts were tested in duplicate, and technical runs were validated using designated internal positive, negative, and extraction controls.

### 2.4. Conventional RT-PCR Amplification of Polymerase Genes

Downstream targeted amplification of the partial PB1 segment 2 and partial PB2 segment 2 was performed using the one-step conventional RT-PCR framework (OneStep RT-PCR kit, Qiagen, Hilden, Germany). Master mixes were prepared in uniform reaction volumes containing: 5 μL of template viral RNA, 5.1 μL of 5× Qiagen OneStep RT-PCR Buffer, 1.1 μL of a 10 mM dNTP blend, 0.4 μL of 50 pmol/μL forward and reverse primer sets, 0.1 μL of RNasin Ribonuclease Inhibitor (40 U/μL, Promega, Madison, WI, USA), and 1.1 μL of Qiagen OneStep RT-PCR Enzyme Mix.

The specific oligonucleotides utilized for mapping segment 2 for PB1 and segment 2 for PB2 were previously developed and validated by Li et al. [37]. The primer designations, sequences, genomic coordinates, and expected molecular weights are listed in Table 2.

The thermal cycling profile was customized on an Rotor-Gene Q 2-Plex HRM thermocycler (Qiagen, Hilden, Germany)using the following kinetic parameters: an initial reverse transcription phase at 50 °C for 30 min (1 cycle), followed by initial PCR activation at 95 °C for 15 min (1 cycle). The amplification phase consisted of 40 cycles of denaturation at 94 °C for 30 s, primer annealing at 52 °C for 30 s (optimized for both PB1 and PB2 designs), and extension at 72 °C for 90 s, culminating in a final elongation step at 72 °C for 10 min. Two independent PCR amplification reactions were performed: one for PB1 segment 2 and one for PB2 segment 2.

The resulting PCR amplicons (~1200 bp) were resolved via horizontal agarose gel electrophoresis to verify diagnostic target bands. Electro-mobility shifts were performed on a 1.5% agarose gel dissolved in 1× TAE/TBE buffer containing 5 μL of ethidium bromide (EtBr, 10 mg/mL). Electrophoretic fractions were prepared by blending 10 μL of the amplified products with 4 μL of tracking loading dye. Electrophoresis was run continuously at 160 V and 1.5 A for 30 min, and target bands were visualized under cross-linking UV transillumination.

### 2.5. Sanger Sequencing Strategy

High-quality, single-band conventional PCR amplicons were outsourced for bidirectional Sanger sequencing to a specialized laboratory infrastructure (Antisel, CEMIA). To maintain downstream sequence continuity, the sequencing reactions utilized the identical forward and reverse primer formulations configured during the conventional RT-PCR screening phase. The PB2 amplification and Sanger sequencing workflow applied in the present study was based on a protocol previously optimized and reported for Romanian avian influenza virus isolates [38]. In contrast to the earlier methodological investigation, the current study expands the molecular characterization to both PB1 and PB2 genes and incorporates mutational and phylogenetic analyses of H5N1 strains collected over an extended surveillance period.

Raw electropherograms and sequence outputs were refined, edited, and assembled using BioEdit Sequence Alignment Editor version 7.2. Following quality curation metrics, both the forward and reverse nucleotide strands were cross-examined to resolve consensus sequences free of technological noise, ambiguous bases, or template impurities. The verified consensus open reading frames for each regional strain were queried against international reference data indexed within the NCBI GenBank database using the Basic Local Alignment Search Tool (NCBI BLAST, web version; National Center for Biotechnology Information, Bethesda, MD, USA).To map specific mutational patterns and adaptive polymorphisms, nucleotide sequences were in silico translated into functional amino acid chains using the standard genetic code.

### 2.6. Phylogenetic and Evolutionary Modeling

Phylogenetic reconstructions were executed through the automated web-based platform https://www.phylogeny.fr using advanced structural modalities. Multiple sequence alignments of the avian influenza polymerase sequences were computed utilizing the MUSCLE algorithm. Structural alignment curation and the removal of poorly aligned or divergent regions were systematically processed through Gblocks v0.91b software.

Evolutionary relationships and topological trees were calculated based on the Maximum Likelihood (ML) criterion using PhyML v3.0 software. Branch support and node reliability were calculated using bootstrap analyses. The final branch configurations and tree topologies were rendered and visually annotated using TreeDyn v198.3 software. Methodological parameters and algorithm calculations for these evolutionary steps were performed according to the guidelines previously described [39,40,41,42,43,44,45].

The sequences selection for the phylogenetic trees took into account that the South–North wild birds migration route was incriminated as the major route of introduction and circulation of AIV H5N1 in Romania and was also the Asian origin of the H5N1 first Romanian isolate [3,11].

Reference sequences were selected from the NCBI GenBank database based on four criteria: (i) highest nucleotide similarity identified by BLAST searches using each Romanian sequence, (ii) sampling during the 2017–2025 H5N1 epidemic period, (iii) geographical representation of the main Eurasian regions involved in recent virus circulation, particularly Central and Eastern Europe, and (iv) inclusion of representative sequences required to preserve the overall phylogenetic context. The resulting dataset was intended to position the Romanian isolates within the contemporary Eurasian evolutionary framework rather than to comprehensively represent global H5N1 diversity.

## 3. Results

### 3.1. Diagnostic Screening and Amplicons Evaluation

Primary laboratory screening via qualitative Real-Time RT-PCR confirmed the presence of highly pathogenic avian influenza (HPAI) H5N1 viral RNA across all 18 investigated biological specimens. The calculated cycle threshold (Ct) values exhibited a broad distribution of genomic loads, ranging from a minimum Ct of 11.90 observed in sample ID 27/683 to a maximum Ct of 38.10 recorded in sample ID 22/10109-4 (Table 3).

Downstream enzymatic processing via conventional one-step RT-PCR targeted an estimated 1200 bp fragment of PB1 segment 2 and second amplified PB2 fragment, encompassing the partial open reading frames.

Visualization of the amplified fractions via horizontal agarose gel electrophoresis demonstrated a direct, stringent correlation between the initial Real-Time RT-PCR viral load and physical amplification efficiency. Robust, high-intensity bands of the expected molecular weight (~1200 bp) were consistently generated from extracts characterized by high genomic concentrations (Ct < 25.00). Conversely, extracts displaying lower viral loads (Ct > 29.00) produced faint diagnostic bands or failed the conventional enzymatic amplification phase entirely.

Samples 16/10175 (Ct 24.03) and 20/10049 (Ct 13.93) had intense electrophoretic bands and were sequenced on PB1, but the sequences had impurities that did not allow for their bioinformatic analysis. Each sample generated amplicons on PB1 and PB2 and were sequenced. Even those samples had weak electrophoretic bands they were subjected to sequencing. The lack of sequences for certain samples even if they were positive by the conventional PCR method is justified by the presence of a significant amount of impurities on the sequence, making it impossible to process them correctly.

Consequently, high-quality bidirectional Sanger sequencing data meeting the strict phylogenetic quality curation metrics were successfully generated for six distinct isolates regarding the PB1 gene (Samples ID 4/10187, 6/12430-2, 9/12430-1, 19/10081, 20/10049, 27/683, and 28/672) and eight distinct isolates regarding the PB2 subunit (Samples ID 4/10187, 6/12430-2, 9/12430-1, 16/10175, 19/10081, 20/10049, 27/683, and 28/672), as systematized in Table 3. The final sequence lengths used for analysis of the PB1 ranged from 765 nucleotides (ID 6/12430-2) to 1116 nucleotides (ID 28/672). The final sequence lengths used for analysis of the PB2 ranged from 762 nucleotides (ID 20/10049) to 1113 nucleotides (ID 19/10081).

### 3.2. Mutational Analysis of the PB1 Segment

Bidirectional Sanger sequencing of the PB1 amplicons yielded definitive consensus sequences for six Romanian isolates (ID 4/10187, 6/12430-2, 9/12430-1, 19/10081, 27/683, and 28/672). Comparative alignment of these sequences against international reference genomes indexed in the NCBI GenBank database revealed a structurally uniform and highly conserved profile across the RNA-dependent RNA polymerase (RdRp) catalytic domain.

**Figure 1:**
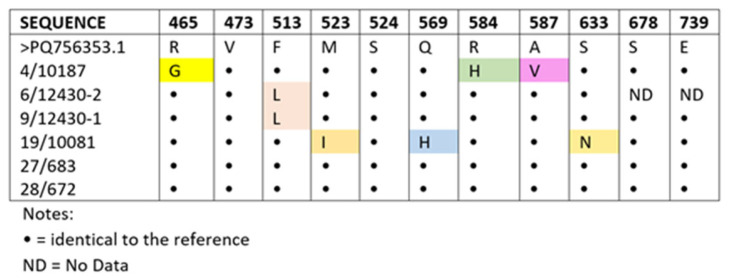
Amino acid substitutions of the partial PB1 protein fragment from Romanian H5N1 avian influenza isolates alongside the reference strain PQ756353.1; colored boxes highlight key polymorphic residues and adaptive target sites.

Molecular screening targeting established amino acid host-adaptation and virulence polymorphisms demonstrated that none of the six sequenced Romanian isolates carried the point mutations 473L, 524T, 678N, or 739V within the PB1 subunit, identifying specific amino acid substitutions at positions 465 (R/G), 513 (F/L), 523 (M/I), 569 (Q/H), 584 (R/H), 587 (A/V), and 633 (S/N) (Figure 1).

### 3.3. Genetic Variation Profiling of the PB2 Segment

The genetic architecture of the partial PB2 gene was successfully resolved for eight regional isolates (ID 4/10187, 6/12430-2, 9/12430-1, 19/10081, 27/683, and 28/672). Targeted molecular screening focused on established mammalian adaptive markers indicated that the primary zoonotic substitution E627K (glutamic acid-to-lysine) was completely absent across all eight Romanian strains, which strictly conserved the wild avian-type glutamic acid (627E) consensus residue. Similarly, the alternative adaptive pathway characterized by the D701N substitution was not detected, with all sequences conserving the baseline aspartic acid at position 701 (701D). The independent mammalian polymorphisms Q591R/K and T271A, as well as the combined G590S/Q591R motifs, were likewise completely absent.

**Figure 2:**
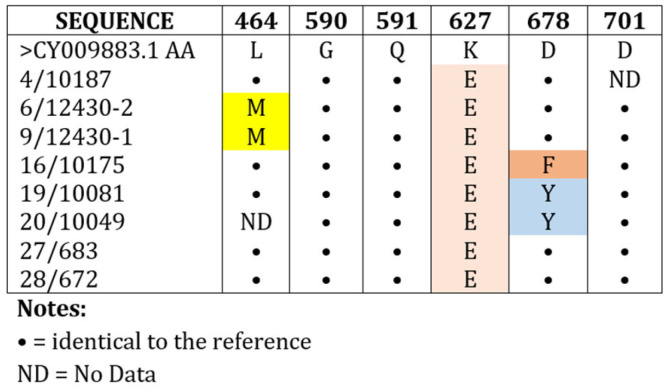
Amino acid substitutions of the partial PB2 protein fragment from Romanian H5N1 avian influenza isolates compared with the reference strain CY009883.1. Colored boxes highlight key polymorphic residues and adaptive target sites.

However, comparative multiple sequence alignment uncovered distinct and recurrent amino acid substitutions within a specific subset of the regional cohort. Notably, five isolates—comprising sample ID 6/12430-2, ID 9/12430-1, ID 16/10175, ID 19/10081 and 20/10049—shared a specific mutational profile characterized by:

- Position 464: A leucine-to-methionine substitution (L464M), mapping onto the hydrophobic core of the mid-structural region in sample ID 6/12430-2 and ID 9/12430-1.
- Position 678: A polymorphic variation where sample ID 16/10175 exhibited a phenylalanine-to-aspartic acid substitution (D678F), whereas sample IDs 19/10081 and 20/10049 presented a tyrosine-to-aspartic acid substitution (D678Y) occurring at the exact localized codon position within the C-terminal 627-domain (Figure 2).

### 3.4. Phylogenetic Topology

**Figure 3:**
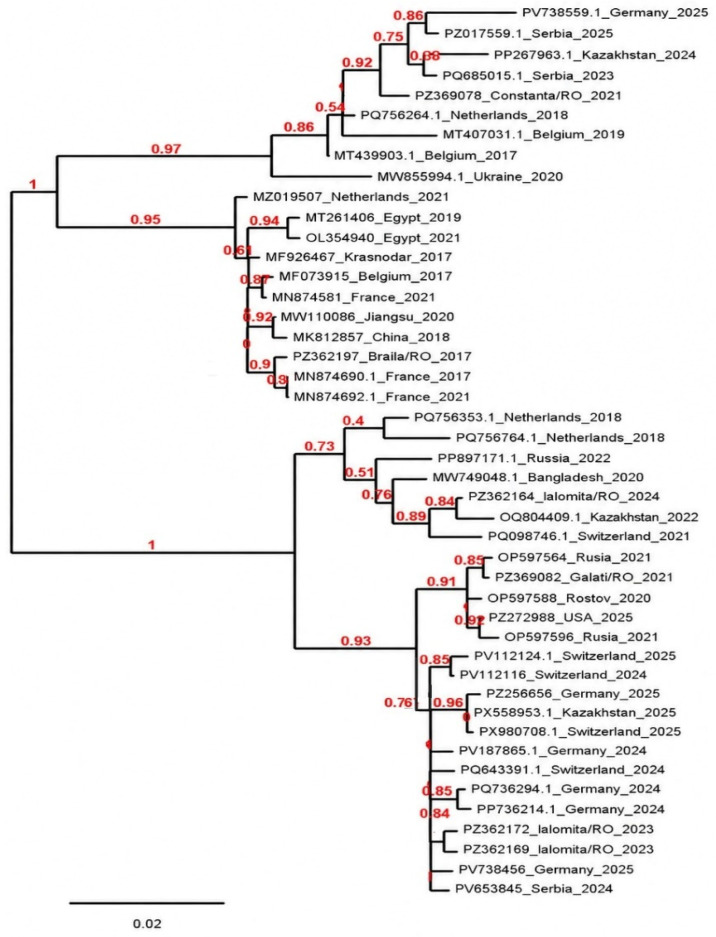
Maximum Likelihood phylogenetic tree of highly pathogenic H5N1 avian influenza strains based on the partial PB1 gene sequences. Numbers at the nodes indicate bootstrap support values.

**Figure 4:**
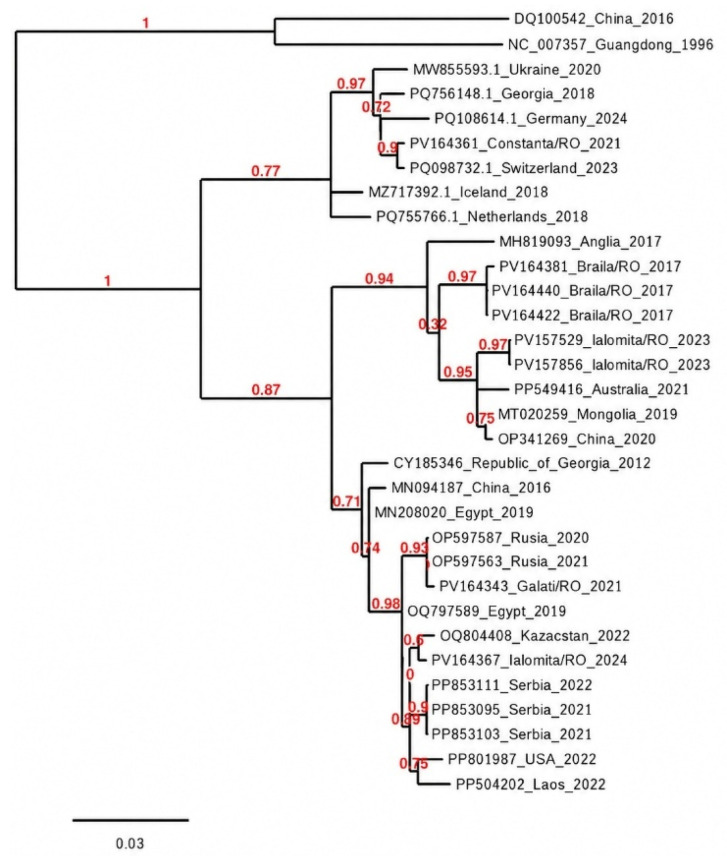
Maximum Likelihood phylogenetic tree of highly pathogenic H5N1 avian influenza strains based on the partial PB2 gene sequences. Numbers at the nodes indicate bootstrap support values.

The phylogenetic trees reconstructed based on the Maximum Likelihood (ML) criterion positioned the partial PB1 and PB2 nucleotide sequences of the Romanian isolates firmly within the established Eurasian avian influenza lineages (Figure 3 and Figure 4).

The topological configuration demonstrated that the Romanian strains did not coalesce into a single, isolated regional cluster but were distributed into distinct sublineages across the evolutionary tree. The contemporary Romanian H5N1 isolates originating from the 2023–2024 epidemic waves (isolates ID 4, 6, and 9) clustered directly together, displaying a high percentage of nucleotide identity and sharing immediate common internal nodes with contemporary H5N1 strains isolated from wild birds and domestic poultry in the Russian Federation and Central Europe. The internal nodes defining these shared phylogenetic branches were characterized by high bootstrap support values, confirming the statistical reliability of the topological layout.

## 4. Discussion

### 4.1. Structural Conservation and Negative Selection Pressure Within the PB1 Subunit

The heterotrimeric RNA-dependent RNA polymerase (RdRp) complex serves as the fundamental engine governing the replication fitness, host adaptation, and evolutionary trajectories of highly pathogenic avian influenza (HPAI) viruses [23]. Within this enzymatic assembly, the PB1 subunit functions as the core catalytic machinery, directly driving nucleotide chain elongation during viral transcription and genome replication [46]. The molecular data generated in this study, characterized via multiple amino acid sequence alignment (Figure 1), underscore a profound structural conservation across the partial PB1 sequences of Romanian H5N1 strains isolated between 2017 and 2024. This rigid structural homogeneity demonstrates that the functional domains of the viral replication machinery are subjected to robust negative selective pressures, as critical deviations within these catalytic motifs frequently compromise viral viability and biological fitness [47].

Despite this baseline conservation, the alignment highlighted a distinct localized variability within specific regions of the PB1 subunit, identifying specific amino acid substitutions at positions 465 (R/G), 513 (F/L), 523 (M/I), 569 (Q/H), 584 (R/H), 587 (A/V), and 633 (S/N). From the literature, it is well established that structural variations localized within the functional windows of 455–460 and 575–590 can directly modulate the spatial configuration of the protein and alter its interaction mechanics with host cellular structures [12,44]. In particular, variations outside the strictly conserved motifs of the PB1 catalytic active site, such as the 587 (V/A) valine-to-alanine shift, can impact the replication efficiency of the ribonucleoprotein (vRNP) complex. Furthermore, mutations like the 633 (S/N) serine-to-asparagine polymorphism may participate in compensatory mechanisms driving viral fitness under variable host environments. Similar structural fluctuations and mutational configurations have been previously described in key molecular surveillance studies mapping the divergence and tissue tropism of H5N1 and H5N8 lineages across different ecosystems [48,49].

Crucially, while these minor polymorphic variations were noted, the absolute absence of primary mammalian-adaptive anchors (such as 473L, 524T, 678N, and 739V) indicates that the circulating Romanian H5N1 strains retain a typical, non-potentiated avian baseline within this internal segment (Figure 1) [50,51]. This high preservation of highly conserved functional epitopes remains a critical focal point, not only for mapping viral evolution but also for the strategic design of targeted antivirals and broad-spectrum universal vaccine formulations [9].

### 4.2. Absence of Primary Zoonotic Anchors: K627E and D701N in the PB2 Gene

The PB2 subunit is widely recognized as one of the primary host-range restriction barriers limiting the efficient transmission and replication of avian influenza viruses within mammalian species [52]. Avian-derived polymerases typically operate sub-optimally at the lower kinetic temperatures (~33 °C) characteristic of the mammalian upper respiratory tract, compared to the higher thermal baseline (~41 °C) typical of the avian respiratory and intestinal systems. To overcome this functional blockade and establish successful infection in mammals, avian strains frequently acquire specific adaptive substitutions in the C-terminal domain of the PB2 protein [53]. Crucially, our molecular characterization demonstrated that the two most prominent mammalian adaptive markers (K627E and D701N) were entirely absent across all successfully sequenced Romanian strains (Figure 2).

The K627E substitution, which exchanges a glutamic acid for a lysine, fundamentally shifts the surface electrostatic charge of the PB2 627-domain, enabling the viral complex to effectively co-opt mammalian host cellular co-factors, such as the nuclear protein ANP32A. Similarly, the alternative D701N substitution enhances viral nuclear import dynamics by increasing the binding affinity between PB2 and mammalian importin-α proteins [54].

The strict preservation of the wild avian-like residues 627E and 701D across the regional cohort confirms that these Romanian H5N1 strains have not undergone the classic, direct pathways of mammalian adaptation. This observation aligns with systemic molecular surveillance frameworks across Eurasian flyways, indicating that while surface glycoproteins fluctuate rapidly, the primary internal zoonotic anchors often remain restricted to wild avian baselines until a stable cross-species spillover event occurs [55,56].

### 4.3. Functional Implications of the Recurrent L464M and D678F/Y Substitutions

Despite the absence of classic mammalian adaptation markers, detailed screening of the PB2 alignment revealed genetic variability characterized by the amino acid substitutions L464M, D678F, and D678Y. These substitutions were identified in different subsets of Romanian H5N1 isolates and therefore represent lineage-specific molecular signatures. The L464M substitution was detected exclusively in isolates ID 6/12430-2 and ID 9/12430-1, both collected from chickens in 2023. In contrast, substitutions at position 678 were identified only in isolates collected during the 2017 outbreak, with D678F detected in isolate ID 16/10175 and D678Y in isolates ID 19/10081 and ID 20/10049 (Figure 2). The recurrent detection of amino acid substitutions at positions 464 and 678 in different groups of Romanian H5N1 isolates indicates that these changes may reflect lineage-specific evolutionary events rather than random mutations. However, because these substitutions occurred in different isolates, host species, and epidemiological years, they should be interpreted as independent molecular signatures of distinct viral lineages.

The L464M substitution (leucine-to-methionine) sits within the mid-structural region of the PB2 architecture. This specific residue alteration maps directly onto the hydrophobic core and can subtly impact the structural flexibility of the vital cap-binding pocket. Biochemical transitions at this codon site may act as stabilizing or compensatory mutations, potentially altering the rate at which the viral ribonucleoprotein (vRNP) complex processes viral mRNA transcripts depending on the host cell environment [57]. While baseline leucine residues are overwhelmingly prevalent in standard wild avian strains, the shift to methionine can modulate overall viral fitness when paired with other internal genetic markers, allowing the polymerase complex to maintain structural and physical stability during cross-species transmission events [58,59].

More significantly, the variations detected at position 678—where both phenylalanine and tyrosine were substituted by aspartic acid (D678F and D678Y)—map directly inside the highly critical C-terminal 627-domain (Figure 2). This specific domain represents the primary biological interface where the virus interacts with host nuclear host factors, most notably the ANP32A protein matrix, to facilitate genome transcription and replication [60]. In the structural topology of the influenza polymerase, position 678 sits in close proximity to the functional loop that governs interaction matrices with the host cells. The substitution of hydrophobic or aromatic residues (F/Y) with a negatively charged, hydrophilic amino acid-like aspartic acid (D) radically alters the localized biochemical microenvironment [61].

Such modifications inside the 627-domain can directly influence the structural flexibility of the domain, potentially modulating its binding affinity to host ANP32A isoforms or altering the overall thermal stability of the polymerase complex. Given that recent global lineages, including the widespread clade 2.3.4.4b, are actively testing diverse alternative mutational configurations to bypass host-range limitations without relying on the classic K627E mutation, these localized adjustments at positions 464 and 678 warrant deep functional investigation [62].

### 4.4. Epidemiology and Flyway Connectivity

Phylogenetic assessment of segment 2 components confirms that the Romanian H5N1 isolates do not represent an isolated, endemic regional reservoir. The Maximum Likelihood topological reconstruction of the partial PB1 gene (Figure 3) and PB2 gene (Figure 4) clearly illustrates that the regional sequences partition into well-defined evolutionary sublineages, reflecting independent introduction events driven by wild migratory waterfowl.

For the PB1 gene, the topology of the phylogenetic tree (Figure 3) highlights the evolutionary relationships between Influenza A virus strains originating from distinct geographical territories, including Romania, Russia, Egypt, France, and China.

The phylogenetic reconstruction indicates that the Romanian PB1 sequences are distributed among several Eurasian sublineages rather than forming a single monophyletic Romanian lineage. Although two well-supported higher-order clusters are observed within the analyzed dataset, these clusters should be interpreted as representing the relationships recovered from the selected reference sequences and not as definitive evidence of independent epidemiological lineages.

The phylogenetic reconstruction indicates that the Romanian PB1 sequences are distributed among several Eurasian sublineages rather than forming a single monophyletic Romanian lineage. The Romanian isolate ID 27/683 with GenBank accession number PZ369078 from Constanța 2021 and isolate ID 4/10187 with GenBank accession number PZ362164 from Ialomița 2024 clustered with strains originating from several European and Asian countries rather than grouping exclusively with each other. Specifically, the isolate ID 27/683 (PZ369078) clustered closely with strains from Serbia (2023), Kazakhstan (2024), Germany (2025), The Netherlands (2018), and Belgium (2019), suggesting that this lineage has been circulating across Europe for several years and has continued to diversify. The relatively high branch support values for this cluster indicate that these relationships are robust and likely reflect a shared evolutionary history. These findings suggest that the clustering of Romanian isolates with viruses from multiple European and Asian countries reflects the broader circulation of H5N1 viruses along migratory bird flyways.

This cross-continental genetic connectivity along flyways is further evidenced by long-term surveillance data from overwintering sites, where related H5 sublineages have historically demonstrated synchronized evolutionary paths between Eurasia and North Africa [63,64,65].

In contrast, the Romanian Ialomița isolates from 2023, ID 9/12430-1 and ID 6/12430-2 (with GenBank accession number PZ362172 and PZ362169, respectively) and from 2024 ID 4/10187 (GenBank accession number PZ362164) were positioned within a different lineage together with strains from Switzerland, Germany, Kazakhstan, Bangladesh, Russia, and Serbia. The close phylogenetic relationship between the Romanian isolates and the recent German (2024–2025) and Swiss (2024–2025) strains suggests that these viruses belong to a lineage that has recently expanded across Europe. Moreover, the close association of the Romanian sequences with isolates from geographically distant regions, including Kazakhstan and Russia, supports the hypothesis of extensive viral dispersal facilitated by animal movements, trade, or migratory wildlife, rather than independent local evolution.

The presence of Romanian sequences in separate subclades, yet deeply tied to Eurasian reference branches, remains fully consistent with historical data demonstrating that avian influenza viruses detected in the Romanian Danubian basin have a predominantly Eurasian genetic origin rather than localized development [66]. These observations align with the well-established ecological paradigm that wild migratory waterfowl operate as the primary global reservoir, continuously facilitating the dissemination and reassortment of Influenza A viruses across vast geographical distances [67].

This pattern of multiple, asynchronous genetic introductions is further validated by the topology of the PB2 phylogenetic tree (Figure 4).

Phylogenetic analysis of the partial PB2 gene showed that Romanian H5N1 HPAI viruses were distributed among several Eurasian sublineages rather than forming a single country-specific lineage, indicating multiple independent introductions into Romania. This pattern is consistent with the epidemiology of clade 2.3.4.4b H5Nx viruses, which are primarily disseminated through migratory birds. The Constanța 2021 isolate ID 27/683 (GenBank accession number PV164361) clustered with viruses from Switzerland (2023), Germany (2024), Georgia (2018), and Ukraine (2020) (bootstrap value of 0.72–0.97), suggesting long-term circulation of this PB2 lineage across Europe. In contrast, the Brăila 2017 isolates ID 16/10175, ID 19/10081 and ID 20/10049 (GenBank accession number PV164381, PV164440, and PV164422) formed a strongly supported monophyletic cluster (bootstrap value of 0.97) closely related to an England 2017 virus, consistent with the European H5N8 epidemic of 2016–2017. Similarly, the Ialomița 2023 isolates ID 6/12430-2 and ID 9/12430-1 (GenBank accession number PV157529 and PV157856) clustered together (bootstrap value of 0.97) and were related to viruses from Australia (2021), Mongolia (2019), and China (2020), reflecting the persistence of widely distributed PB2 lineages within the global Goose/Guangdong H5 gene pool rather than direct epidemiological links. The Galați 2021 isolate ID 28/672 (GenBank accession number PV164343) grouped with Russian viruses from 2020 to 2021 (bootstrap value of 0.93), indicating an eastern Eurasian lineage, whereas the Ialomița 2024 isolate ID 4/10187 (GenBank accession number PV164367) clustered with viruses from Kazakhstan (2022) and Serbia (2021–2022) (bootstrap value 0.89–0.90), supporting circulation of closely related H5N1 viruses along the Black Sea–Mediterranean and Central Asian migratory flyways. Overall, the PB2 phylogeny indicates that Romanian HPAI viruses originated from repeated introductions of genetically distinct Eurasian lineages rather than persistent local evolution. Their close relationships with viruses from Europe and Asia highlight the extensive transboundary circulation of clade 2.3.4.4b viruses and emphasize the role of migratory birds in shaping the molecular epidemiology of HPAI in Romania.

Ultimately, the tree topologies and high bootstrap distributions across both polymerase genes confirm a highly robust phylogenetic architecture. These results strongly emphasize the vital necessity of maintaining active, continuous molecular surveillance and deep phylogenetic characterization to monitor the evolution of Influenza A viruses and ensure the early identification of emerging variants with elevated epidemic or zoonotic potential [68,69].

An important epidemiological implication of these phylogenetic findings is that Romanian H5N1 viruses do not represent a single endemic lineage. Instead, their distribution across multiple PB1 and PB2 subclades strongly supports repeated independent introductions, most likely associated with migratory bird flyways connecting Romania with other Eurasian regions. This pattern is consistent with the current epidemiology of clade 2.3.4.4b viruses circulating throughout Europe.

### 4.5. Study Limitations

The present study has several limitations that should be considered when interpreting the findings. Although eighteen H5N1-positive samples were analyzed, high-quality sequence data were successfully obtained for only six PB1 and eight PB2 fragments, limiting the statistical power and the ability to infer broader evolutionary trends. Furthermore, only partial gene fragments were characterized rather than complete viral genomes, preventing comprehensive assessment of reassortment events and genome-wide evolutionary dynamics. The phylogenetic analyses presented in this article should be interpreted as providing evolutionary context rather than exhaustive reconstruction of transmission pathways.

Finally, the biological significance of the identified amino acid substitutions remains to be experimentally validated through functional studies.

## 5. Conclusions

This study provides a molecular characterization of the PB1 and PB2 genes of Romanian H5N1 viruses collected between 2017 and 2024. The analyzed sequences showed a high degree of conservation, with no evidence of the major mammalian-adaptation markers K627E or D701N. Phylogenetic analyses demonstrated that Romanian isolates are distributed across multiple Eurasian subclades, supporting repeated introductions rather than the persistence of a single endemic lineage. Although based on a limited number of partial sequences, these findings highlight the value of continued molecular surveillance for monitoring the evolution and spread of H5N1 viruses in Romania.

## Figures and Tables

**Table 1:** Epidemiological and clinic-pathological metadata of the analyzed highly pathogenic avian influenza (HPAI) H5N1 samples.

No.	Sample ID.	County/Country	Year	Species	Sample Type
1.	4/10187	Ialomita/Romania	2024	Swan	Multi-organ homogenate (brain, trachea, lungs, spleen, liver, pancreas, kidneys, intestine)
2.	6/12430-2	Ialomita/Romania	2023	Hen	Multi-organ homogenate (brain, trachea, lungs, spleen, liver, pancreas, kidneys, heart)
3.	9/12430-1	Ialomita/Romania	2023	Hen	Multi-organ homogenate (brain, trachea, lungs, spleen, liver, pancreas, kidneys, intestine)
4.	14/10215	Braila/Romania	2017	Hen	Lung tissue
5.	15/10233	Braila/Romania	2017	Hen	Multi-organ homogenate
6.	16/10175	Braila/Romania	2017	Hen	Multi-organ homogenate
7.	17/19215	Braila/Romania	2017	Hen	Multi-organ homogenate
8.	18/10111	Braila/Romania	2017	Hen	Multi-organ homogenate
9.	19/10081	Braila/Romania	2017	Swan	Multi-organ homogenate
10.	20/10049	Braila/Romania	2017	Swan	Multi-organ homogenate
11.	21/10109-5	Braila/Romania	2017	Hen	Brain tissue
12.	22/10109-4	Braila/Romania	2017	Hen	Multi-organ homogenate
13.	23/10109-2	Braila/Romania	2017	Hen	Spleen tissue
14.	24/10108	Braila/Romania	2017	Hen	Lung tissue
15.	25/101110	Braila/Romania	2017	Hen	Multi-organ homogenate
16.	26/10081	Braila/Romania	2017	Hen	Multi-organ homogenate
17.	27/683	Constanta/Romania	2021	Swan	Multi-organ homogenate
18.	28/672	Galati/Romania	2021	Swan	Multi-organ homogenate

**Table 2:** Oligonucleotide primer sequences and molecular parameters utilized for the conventional RT-PCR amplification of PB1 and PB2 gene segments.

Target Gene	Primer Name	Sequence Direction (5′ → 3′)	Amplicon Size (bp)	Reference
PB1	PB1-1124F	ARATACCNGCAGARATGCT	~1200	[37]
	Bm-PB1-2341R	ATATCGTCTCGTATTAGTAGAAACAAGGCATTT		
PB2	PB2-1105F	TAYGARGARTTCACAATGGT	~1200	
	Bm-PB2-2341R	ATATGGTCTCGTATTAGTAGAAACAAGGTCGTTT		

Number in the name of the primer indicates the nucleotide position of the first base in the target sequence.

**Table 3:** Real-Time RT-PCR cycle threshold (Ct) values, conventional PCR outcomes, and NCBI GenBank accession numbers for the analyzed HPAI H5N1 segments.

No.	Sample ID.	Real-Time RT-PCR Ct Value	Conventional PCR Result	GenBank Accession PB1	GenBank Accession PB2
1	4/10187	17.61	Positive	PZ362164.1	PV164367.1
2	6/12430-2	15.17	Positive	PZ362169.1	PV157529.1
3	9/12430-1	12.92	Positive	PZ362172.1	PV157856.1
4	14/10215	30.12	Weak Positive	—	—
5	15/10233	32.11	Negative	—	—
6	16/10175	24.03	Positive	—	PV164381.1
7	17/19215	30.5	Weak Positive	—	—
8	18/10111	25.6	Weak Positive	—	—
9	19/10081	21.6	Positive	PZ362197.1	PV164440.1
10	20/10049	13.93	Positive	—	PV164422.1
11	21/10109-5	28.81	Weak Positive	—	—
12	22/10109-4	38.1	Negative	—	—
13	23/10109-2	29.73	Weak Positive	—	—
14	24/10108	31.63	Negative	—	—
15	25/101110	30.04	Negative	—	—
16	26/10081	37.61	Negative	—	—
17	27/683	11.9	Positive	PZ369078.1	PV164361.1
18	28/672	13.11	Positive	PZ369082.1	PV164343.1

## Data Availability

The nucleotide sequences generated in this study have been deposited in GenBank under accession numbers PZ362164.1–PZ369082.1 and PV157529.1–PV164422.1. Additional data supporting the findings of this study are available from the corresponding authors upon reasonable request.
